# Feasibility of Long-Term Physical Activity Measurement With a Wearable Activity Tracker in Patients With Axial Spondyloarthritis: 1-Year Longitudinal Observational Study

**DOI:** 10.2196/68645

**Published:** 2025-05-07

**Authors:** Emil Eirik Kvernberg Thomassen, Anne Therese Tveter, Inger Jorid Berg, Eirik Klami Kristianslund, Andrew Reiner, Sarah Hakim, Laure Gossec, Gary J Macfarlane, Annette de Thurah, Nina Østerås

**Affiliations:** 1 Centre for Treatment of Rheumatic and Musculoskeletal Diseases, Diakonhjemmet Hospital, Diakonveien 12, Oslo, 0370, Norway, 47 95252216; 2 Faculty of Medicine, University of Oslo, Oslo, Norway; 3 Department of Rehabilitation Science and Health Technology, OsloMet – Oslo Metropolitan University, Oslo, Norway; 4 Oslo Centre for Biostatistics & Epidemiology, Oslo University Hospital, Oslo, Norway; 5 INSERM, Institut Pierre Louis d’Epidémiologie et de Santé Publique, Sorbonne Université, Paris, France; 6 Rheumatology Department Pitié-Salpêtrière Hospital, Assistance Publique – Hôpitaux de Paris, Paris, France; 7 Aberdeen Centre for Arthritis and Musculoskeletal Health (Epidemiology Group), University of Aberdeen, Aberdeen, United Kingdom; 8 Department of Rheumatology, Aarhus University Hospital, Aarhus, Denmark; 9 Department of Clinical Medicine, Aarhus University, Aarhus, Denmark

**Keywords:** physical activity, wearables, axial spondyloarthritis, activity trackers, feasibility

## Abstract

**Background:**

Using wearable activity trackers shows promise in measuring physical activity in patients with axial spondyloarthritis (axSpA). However, little is known regarding the feasibility of long-term use.

**Objectives:**

This study aimed to explore the feasibility of recording physical activity using a wearable activity tracker and describe wear-time patterns among patients with axSpA.

**Methods:**

Data from a randomized controlled trial (NCT: 05031767) were analyzed. Patients with axSpA and low disease activity were recruited from an outpatient clinic and asked to wear a Garmin vívosmart 4 activity tracker for 1 year. The activity tracker measured steps and heart rate. Trial feasibility (eligibility, inclusion rate, and patient characteristics), technical feasibility (data recorded, tracker adherence, ie, days worn, and missing data), and operational feasibility (synchronization reminders and tracker replacements) were analyzed. Tracker adherence was calculated as the percentage of recorded minutes of the maximum possible minutes. Unsupervised hierarchical clustering was used to explore tracker wear-time patterns.

**Results:**

Of the 160 patients screened, 75 (47%) agreed to use the tracker and 64 (85%) were analyzed (11 had insufficient data). The median activity tracker adherence over 1 year was 66% (IQR 30‐86). There was 30% missing step and 0.01% heart rate data in the physical activity dataset. A median of 18 (IQR 9‐25) reminders per patient to synchronize activity data were distributed. Analysis of wear-time patterns resulted in 3 groups: Adherent (33/64, 51% of patients), Minimal Use (17/64, 27%), and Intermittently adherent (14/64, 22%).

**Conclusions:**

Trial feasibility was low, while technical and operational feasibility were acceptable. Only 51% of the patients were highly adherent. Activity trackers, though trendy, have low to moderate feasibility over 1 year in patients with axSpA. Automated synchronization and adherence barriers should be further explored.

## Introduction

Recent technological advancements enable novel ways of managing patients with chronic disease by using wearable devices, such as activity trackers [1]. In addition to measuring physical activity, wearable activity trackers can act as motivators to increase levels of physical activity [2-4]. axSpA is a chronic inflammatory joint disease, primarily characterized by sacroiliitis, back pain, and stiffness [5]. First-line management includes nonsteroidal anti-inflammatory drugs and regular exercise [6]. However, there are indications that patients with axSpA engage in lower levels of physical activity compared with healthy people [7-9] and that they report more barriers to engaging in physical activity compared to controls [10]. Identifying physically inactive patients allows for optimized treatment by supporting patients’ self-management of their disease and potentially enhancing exercise adherence [11,12]. The passive collection of data using wearables has been highlighted as a goal within remote monitoring in rheumatology, as it may ease the monitoring of disease activity besides using electronic patient-reported outcomes [13,14]. In addition, continuous measurement with an activity tracker has the potential to provide further insight into how the physical activity levels of patients with axSpA are affected by their disease [15-17].

In other patient groups, such as osteoarthritis and gout, research has shown low to acceptable adherence to the use of activity trackers during 3- and 6-month periods, with declining adherence toward the end of the studies [18,19]. However, further investigation is warranted, given the lack of evidence on the feasibility and adherence to long-term use of wearable activity trackers among patients with axSpA [20-24].

The aim of this study was to explore the trial, technical, and operational feasibility of measuring physical activity using commercially available wearable activity trackers over 1 year among patients with axSpA. Second, the study aimed to analyze wear-time patterns and compare patients’ demographic and clinical characteristics between the wear-time clusters.

## Methods

### Study Design and Setting

This study includes a post hoc analysis of a randomized controlled trial, titled Remote Monitoring in Specialist Health Care Study (ReMonit, ClinicalTrials.gov: NCT05031767). The primary results of the trial are reported elsewhere [25]. The ReMonit Study was a 3-armed randomized controlled trial comparing remote monitoring and patient-initiated care to usual care (prescheduled regular hospital visits) among patients with axSpA [26]. Patients were recruited from an outpatient clinic at Diakonhjemmet Hospital, Oslo, Norway, and randomized 1:1:1 ratio to receive usual care, remote monitoring, or patient-initiated care. Patients randomized to the remote monitoring and patient-initiated groups were asked to use the activity tracker for 1 year. This was a commercially available activity tracker (Garmin vívosmart 4). Physical activity data recorded by the vívosmart 4 was wirelessly transferred manually by patients each week via Garmin Software Development Kit to the MyDignio app [27].

### Patients

In the ReMonit study, patients with axSpA with low disease activity (Axial Spondyloarthritis Disease Activity C-Reactive Protein Score (ASDAS-CRP) <2.1) and stable treatment with a tumor necrosis factor inhibitor (TNFi) over the past 6 months were included [26]. Since we used the Garmin Software Development Kit for the present study, patients who already used a Garmin device and the Garmin Connect app could not be included, as this interfered with synchronization of data to the MyDignio app. In addition, due to privacy regulations, direct downloading of data from the patients’ private Garmin devices was not allowed. Therefore, patients could not use their private devices in the present study.

### Data Collection

At baseline, patients completed a digital questionnaire including age, sex, education level, and working status. BMI was calculated based on self-reported body height and weight, and information on years since diagnosis of axSpA was obtained from their medical records. The patients completed the recommended disease-specific questionnaires [28] such as Bath ankylosing spondylitis disease activity index (BASDAI) (0‐10, 10 being the worst score), Bath ankylosing spondylitis functional index (BASFI) (0‐10, 10 being the worst score), and patient global assessment (0‐10, 10 being the worst score) [29,30]. The ASDAS-CRP was calculated based on patients’ self-reported disease activity-related questions and measurement of C-reactive protein [31]. The Work Productivity and Activity Impairment item 6 (WPAI) (0‐10, 10 being the worst score) was used for measuring the impact of the disease on patients’ everyday life [32]. In addition, eHealth literacy was measured by 4 scales from the eHealth Literacy Questionnaire [33]: Using technology to process health information (Scale 1), Ability to actively engage with digital services (Scale 3), Feel safe and in control (Scale 4), and Motivated to engage with digital services (Scale 5). For measuring self-reported physical activity and exercise, we used 3 items from a population-based study measuring the frequency, duration, and intensity of exercise [34]. We then further stratified the patients into 3 groups based on whether the level of physical activity was below recommended (below 150 min of moderate-intensity exercise per week), at recommended (150 moderate or 60 vigorous minutes each week), or above recommended [35].

### Feasibility

To assess the feasibility of using activity trackers for measuring physical activity, we categorized feasibility into 3 novel subcategories inspired by a previous feasibility study on activity trackers [18].

#### Trial Feasibility

This included the inclusion rate (number of patients included), eligibility of patients (characteristics of patients who declined and those included), proportion of patients with recorded data, and differences in patient characteristics between patients in the clusters. For evaluating the trial feasibility, we considered the inclusion rate and proportion of patients with recorded data corresponding to <50% as low, 50‐70% as moderate, and >70% as high.

#### Technical Feasibility

This included the number of physical activity minutes recorded by patients, adherence to wearing the activity tracker, and missing data on steps or heart rate. Adherence to the use of the activity tracker was defined and calculated as the number of minutes with recorded data by each patient (per minute) divided by the total number of minutes during daytime (16 h; 348,000 min × 100). Adherence was classified as either low (<50%), moderate (50‐70%) or high (>70%). An acceptable level of missing data was determined to be below 40% [36].

#### Operational Feasibility

This included the number of automatic reminders sent to the patients (in comparison to the total maximum possible number of 3900 reminders, eg, 52 weeks × 75 patients), and number of replaced activity trackers during the 1-year period. Equal to or less than a median of 26 reminders per patient (eg, in 50% of the weeks) and<15% replacements of activity trackers in the study sample were deemed acceptable.

### Measurement of Physical Activity

The patients received oral and written instructions stating that the activity tracker should be worn on the non-dominant wrist for at least 10 hours a day. It should not be worn on the outside of garments but could be used while swimming and showering. Instructions were also given regarding charging, connecting, and synchronizing the device with the app. Contact information for the study team was provided in the event of technical issues.

The Garmin vívosmart 4 activity tracker (Olathe) was integrated into the MyDignio app by the company Dignio. Physical activity data were recorded at a minute level, measuring both steps per minute and average heart rate during the concurrent minute. The Garmin vívosmart 4 uses an accelerometer for measurement of physical movement and activity and a photoplethysmography sensor for measuring heart rate [37].

In order to reduce data noise from the activity tracker, a filter was applied by Dignio, which filtered out heart rate values below 20 beats per minute. The native Garmin motivational messages and notifications were muted. Patients were instructed to weekly synchronize physical activity data manually from the device using the MyDignio app due to limited internal memory of the activity tracker. If patients failed to synchronize in time, the MyDignio app automatically sent a push notification as a reminder. If patients still did not synchronize after receiving the push notification, the study team followed up with an SMS text message reminder. The number of manual SMS text message reminders sent out was not registered. Any technical issues regarding app connectivity or the activity tracker were primarily resolved by the study team, or if necessary, by developers at Dignio.

### Data Analyses

Median values with IQR or mean values with SD were used for describing the demographical and clinical variables. Data regarding the trial, technical, and operational feasibility were described by either percentage, mean with SD, or median with IQR. Differences between patients agreeing to use a wearable compared to those declining were assessed by the Mann-Whitney *U* test or Student’s *t*-test.

To assess wear-time patterns based on the data returned from the activity tracker, we used the hourly level data, which were summed up on a weekly level. Each patient’s first week of recording was assigned as their baseline week. Unsupervised hierarchical clustering using the pheatmap package in RStudio was used for assessing different wear-time patterns based on the number of hours of physical activity data recorded [38]. The optimal numbers of predefined clusters were based on the visual inspection of the clustered heatmap and internal validation from the R-package clValid [39]. The cluster groups were named according to their observable patterns that occurred within each group. Patient characteristics in the clustering groups for wear-time patterns were compared and tested for significant differences using the Kruskal-Wallis test, and Dunn test if the initial Kruskal-Wallis test had a *P*-value below 0.05. The Fisher exact test was used for testing differences in the distribution of sexes between the groups. Preparation and analysis of data were conducted using RStudio and Stata (version 18.0; StataCorp LLC).

### Ethical Considerations

The study was conducted according to the Helsinki Declaration. All patients provided written consent to participate and were informed that they could stop using the activity tracker at any given time during the study. Data were de-identified and stored at a secure research server. The Regional Committees for Medical and Health Research Ethics South-Eastern Norway approved the study (ref: 229187).

### Patient and Public Involvement

In total, 2 patient research partners were involved in the planning of the study. They also pilot-tested the activity tracker and gave feedback on the instructions to the patients and on the study logistics. One of the patient research partners (SH) also contributed to analyzing and discussing the results and is a co-author of this manuscript.

## Results

### Trial Feasibility

**Figure 1.**
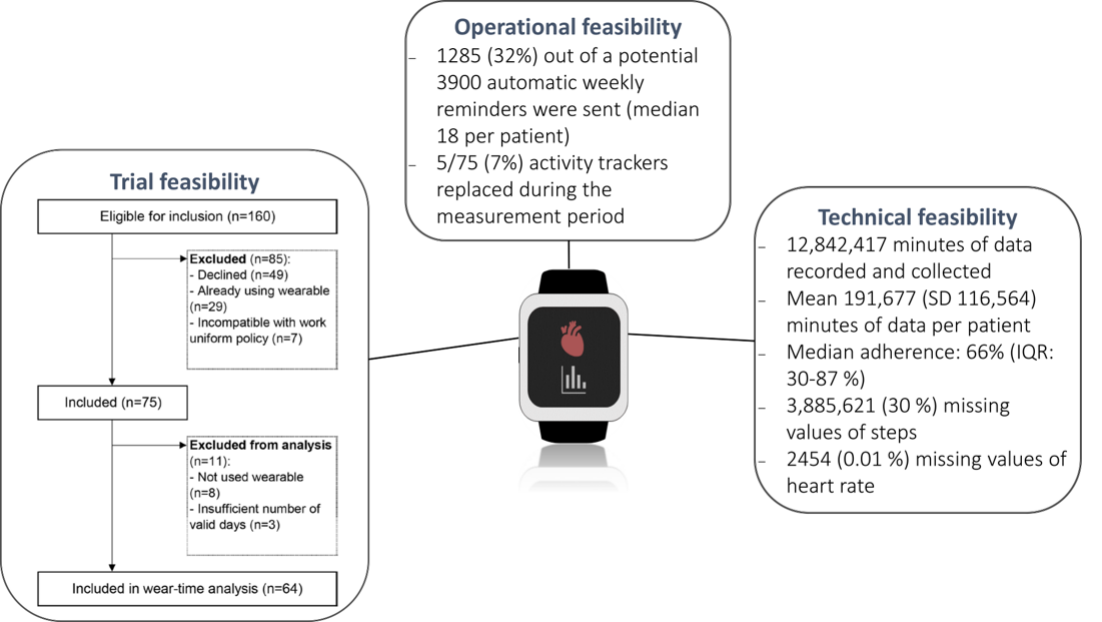
Trial, technical, and operational feasibility of using a wearable activity tracker to record physical activity over 1 year.

A total of 75 (47%) out of 160 patients agreed to use the activity tracker for 1 year and were included in this study (Figure 1). Among the 85 non-participating patients, 49 (31%) declined to wear an activity tracker, 29 (18%) were already users of a Garmin activity tracker device, and 7 (4%) could not wear an activity tracker due to uniform regulations at their workplace (Figure 1). Baseline characteristics were mostly similar between patients who agreed and those who declined to use the activity tracker, with minor numerical variances in self-reported physical activity levels (Table 1). Patients who declined had significantly lower eHealth literacy scores compared with those who agreed to use the activity tracker, but the numeric differences in mean scores were small (Table 1).

**Table 1.** Baseline characteristics of the total sample of patients asked to use the wearable activity tracker for 1 year and subsamples.

Characteristics	Total sample asked to use activity trackers (N=160)	Agreed to use the activity tracker (n=75)	Declined to use the activity tracker (n=49)	Ineligible^a^ to use the activity tracker (n=36)
Age, years, median (IQR); min-max	42 (34‐51); 22‐70	43 (34‐53); 24‐70	42 (39‐50); 22‐68	41 (32‐50); 24‐63
Males, n (%)	123 (77)	57 (76)	39 (77)	28 (78)
Education level, n (%)				
Primary level	33 (21)	19 (25)	9 (18)	5 (14)
University level	127 (79)	56 (75)	40 (82)	31 (86)
Working status, n (%)				
Full-time paid work	126 (79)	59 (79)	39 (80)	28 (78)
Age retired or disability pension	13 (8)	9 (12)	3 (6)	1 (3)
Other^b^	21 (13)	7 (9)	7 (14)	7 (19)
BMI (kg/m^2^), median (IQR)	24.9 (22.8‐27.2)	25.2 (23.0‐27.5)	24.6 (23.1‐27.4)	23.8 (21.8‐26.1)
ASDAS-CRP^c^, median (IQR)	0.9 (0.6‐1.4)	0.9 (0.6‐1.5)	0.9 (0.6‐1.2)	0.8 (0.6‐1.3)
BASDAI^d^, median (IQR)	1.0 (0.3‐2.0)	1.2 (0.5‐2.3)	1.0 (0.7‐2.0)	0.6 (0.1‐1.8)
Fatigue, median (IQR)	1.0 (0.0‐3.0)	2.0 (1.0‐4.0)	2.0 (0.0‐3.0)	1.0 (0.0‐2.0)
Morning stiffness, median (IQR)	1.0 (0.0‐2.5)	1.0 (0.0‐2.5)	1.0 (0.0‐2.0)	1.0 (0.0‐1.5)
PGA^e^, median (IQR)	1 (0.0‐2.0)	1.0 (1.0‐3.0)	1.0 (1.0‐2.0)	1.0 (0.0‐2.0)
BASFI^f^, median (IQR)	0.3 (0.0‐1.2)	0.4 (0.1‐1.7)	0.2 (0.0‐0.9)	0.1 (0.0‐0.8)
Years since axSpA diagnosis, median (IQR)	12 (6‐20)	12 (6‐23)	12 (8‐19)	10 (5‐17)
Self-reported physical activity level^g^, n^h^ (%)				
Below recommended	10 (11)	3 (7)	5 (21)	2 (11)
Recommended	25 (28)	12 (26)	7 (29)	6 (33)
Above recommended	53 (60)	31 (67)	12 (50)	10 (56)
eHealth literacy, mean (SD)				
eHLQ Scale 1	3.3 (0.5)	3.3 (0.5)	3.1 (0.6)	3.3 (0.6)
eHLQ Scale 3	3.6 (0.4)	3.6 (0.4)	3.5 (0.5)	3.5 (0.6)
eHLQ Scale 4	3.4 (0.4)	3.4 (0.4)	3.3 (0.4)	3.4 (0.5)
eHLQ Scale 5	3.3 (0.5)	3.4 (0.4)	3.1 (0.5)	3.2 (0.6)

a Ineligible: due to uniform regulations at work place or already owning a Garmin activity tracker.

b Other: receiving social benefits, on sick leave, student/housekeeping, part-time work and unemployed, ASDAS-CRP: Ankylosing spondylitis Disease Activity Score, BASDAI: Bath ankylosing spondylitis disease Activity Index (0-10, 10 being worst), BASFI: Bath ankylosing spondylitis functional index (0-10, 10 being worst).

c ASDAS-CRP: Axial Spondyloarthritis Disease Activity C-Reactive Protein Score.

d BASDAI: Bath ankylosing spondylitis disease activity index.

e PGA: patient global assessment.

f BASFI: Bath ankylosing spondylitis functional index.

g Self-reported using the HUNT physical activity questionnaire based on World health organization recommendations on physical activity.

h n: lower number of participants due to missing.

i eHLQ: eHealth literacy Questionnaire (0-4, 4 being best): eHLQ Scale 1: Using technology to process health information, eHLQ Scale 3: Ability to actively engage with digital services, eHLQ Scale 4: Feel safe and in control, eHLQ Scale 5: Motivated to engage with digital services.

### Technical Feasibility

Among the 75 patients who agreed to use the activity tracker, 64 (85%) had valid physical activity data and a median adherence to use the activity tracker of 66% (IQR 30‐86) (Figure 1). Over the 1-year measurement period, 8 out of 75 patients never recorded any data. In total, 3 patients returned less than 1000 minutes of data recorded (corresponding to approximately a total of 16 h of physical activity data) and were subsequently excluded from the wear-time pattern analyses during aggregation of data (Figure 1). A total of 12 million datapoints were returned, corresponding to a mean of 191,677 (SD 116,564) minutes of data per patient (Figure 1). Analyses showed that the proportion of missing data was higher for the steps (30%) compared to heart rate data (0.01%).

### Operational Feasibility

To remind patients to synchronize the activity tracker data, a total of 1285 (32%) out of a potential 3900 automatic push notifications were distributed, with a median of 18 (IQR 9‐25) notifications per patient. In total, 5 activity trackers required replacement during the study, of which 4 were caused by connectivity issues with the app and one due to a loss of the activity tracker (Figure 1).

### Wear-Time Patterns

**Figure 2.**
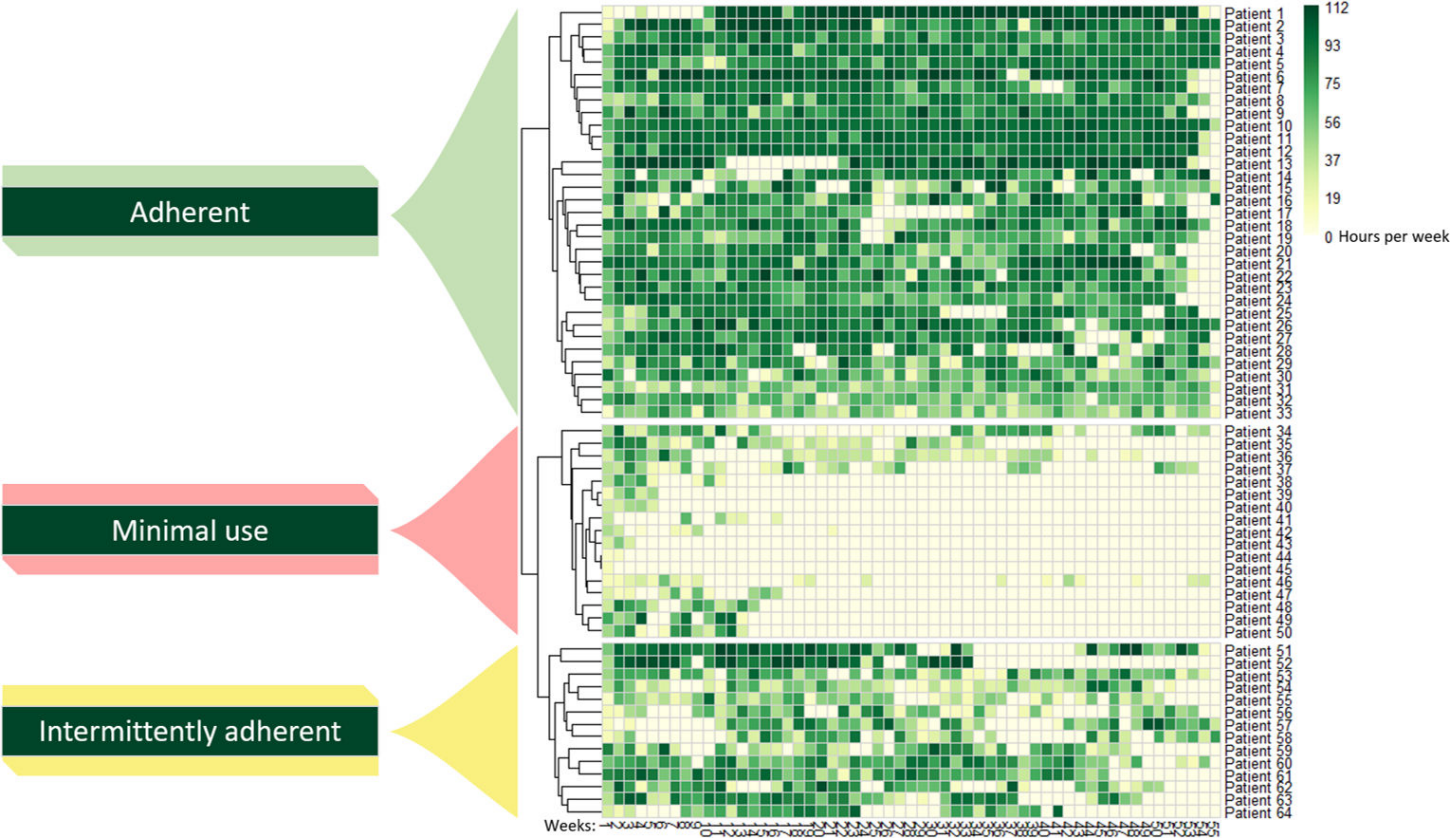
Hierarchical clustered heatmap containing 3 cluster based on wear-time patterns, expressed as hours of physical activity data per week per patient (n=64). The x-axis shows the weeks for each patient who recorded data, with the y-axis showing the number of hours of recorded data per week. Dendrograms at the left show the clustering.

Data on wear time were grouped in clusters according to the visual wear-time patterns, and 3 cluster groups were found to be the optimal number of clusters. The 3 cluster groups were categorized as “Adherent” (n=33), “Minimal use” (n=17) and “Intermittently adherent” (n=14) according to their visible wear-time patterns (Figure 2). The patients included in the Adherent group had a steady recording of physical activity data, the Minimal use group showed longer periods of no recorded data, and the Intermittently adherent group presented with a notably larger variation in the amount of recorded data (Figure 2). We found that the Adherent group had a median of 85% (IQR 72‐89) adherence to the use of the activity tracker, while the Minimal use and Intermittently adherent groups had lower adherence with 7% (IQR 4‐16) and 49% (IQR 43‐59), respectively (Table 2).

**Table 2.** Age, sex, self-reported physical function, impact on daily activities, and disease activity across 3 cluster groups with different wear-time patterns.

	Adherent (n=33)	Minimal use (n=17)	Intermittently adherent (n=14)
Age, years median (IQR)	46 (42‐55)	35 (32‐50)	40 (33‐53)
Sex, males n (%)	23 (70)	16 (94)	10 (71)
BASFI^a^, median (IQR)	1.1 (0.2‐1.9)	0.2 (0.0‐0.4)	0.7 (0.3‐1.9)
WPAI^b^, median (IQR)	1.0 (0.0‐3.0)	0.0 (0.0‐1.0)	1.0 (0.0‐2.0)
ASDAS^c^, median (IQR)	1.2 (0.7‐1.8)	0.8 (0.6‐1.2)	1.1 (0.7‐1.7)
Adherence^d^, % median (IQR)	85 (72‐89)	7 (4-16)	49 (43‐59)

a BASFI: Bath ankylosing spondylitis functional index (0-10, 10 being worst).

b Work Productivity and Activity Impairment, Ability to perform daily activities (WPAI Item 6, NRS 0-10, 10 being worst).

c ASDAS-CRP: Ankylosing spondylitis Disease Activity Score.

d Adherence to use the activity tracker was calculated as data returned divided by maximum amount of day time data.

The Minimal use group had a significantly (*P*=.019) lower median age (35 y, IQR 32‐50) compared to the Adherent (46 y, IQR 42‐55) and Intermittently adherent groups (40 y, IQR 33‐53) (Table 2). There were nonsignificant between-group differences (*P*=.107) in the proportion of sex, with 23 out of 33 (70%) males in the Adherent group, 16 out of 17 (94%) in the Minimal use group, and 10 out of 14 (71%) in the Intermittently adherent group. There were no differences between the groups in disease activity, self-reported physical function, or the impact on daily activities.

## Discussion

### Principal Findings and Comparison With Previous Works

This study explored the feasibility and wear-time pattern of long-term use of a commercially available activity tracker over 1 year among patients with axSpA. The results indicated a low trial feasibility and acceptable technical and operational feasibility. In total, 3 different cluster groups were identified, with the largest group demonstrating adherence to using the wearable activity tracker.

In the assessment of trial feasibility, we found a low inclusion rate for the activity tracker, with less than half of the 160 eligible patients agreeing to wear the activity tracker for 1 year. This might indicate that the willingness to use a wearable activity tracker over a long period may not be present among the majority of patients with axSpA with low disease activity. However, we believe that a higher inclusion rate could have been achieved if direct data downloads from patients’ private Garmin devices had been possible. Implementing such solutions may require specially adapted software and the use of “proxy-users” [40], potentially leading to complex data management.

We observed that a small proportion of patients either did not record any data or recorded less data than the cutoff for a valid day (>10 hours). An earlier study on patients with gout reported similar findings, showing that 33 of 44 patients had valid data, and 40% of the total data was missing [19]. Missing data, when measuring at this high-level granularity, are expected but yet remain a challenge considering the use of activity trackers in clinical settings [41]. A possible reason for the high proportion of missing steps data might be due to differences between Android and iOS phones in the interpretation of the step data, where Android phones misinterpreted inactivity as missing data instead of providing the numerical value of 0. The low number of missing heart rate data may be due to the filter that deleted observations of heart rates below 20 beats per minute, possibly resulting in a skewed representation of the number of missing heart rate data. Additionally, the discontinuation of using the activity tracker has been discussed in earlier studies, showing that perceived usefulness and inaccuracy of data could be a reason for discontinuing the use of activity trackers among patients with osteoarthritis and for healthy people [18,42]. Patients’ perception of data inaccuracy may also play a role in our study. Since we only recorded steps and heart rate as measures of physical activity, we effectively limited our recordings to step-related activities, thereby excluding popular Norwegian activities such as bicycling, cross-country skiing, and swimming.

The operational feasibility showed that a median of 18 reminders were sent out per patient, indicating a fairly low number based on the fact that reminders were sent out on a weekly level. It was also observed that the majority of tracker replacements were due to connectivity issues, which could lead to longer periods without recording physical activity.

The hierarchical clustering analysis showed that the largest cluster group was patients who showed adherence to using the activity tracker. Since this represents 33 out of a possible 160 patients, it could be argued that using activity trackers is only feasible for this specific group of patients. The between-group comparisons of patient characteristics revealed numerically small differences between the 3 wear-time cluster groups regarding age and proportion of sex. However, all 3 groups had a small sample size, which limits trust in the between-group comparisons.

Considering the potential benefits of physical activity monitoring, activity trackers may prove valuable in guiding and motivating patients to engage in higher levels of physical activity [2]. Longitudinal data on physical activity can potentially offer new insights into how axSpA impacts patients’ activity levels and can aid in developing targeted exercise and physical activity interventions. The collection of longitudinal physical activity may potentially support physiotherapists and other health care professionals in prescribing lifestyle interventions for patients with axSpA [2]. However, challenges such as low adherence and technical issues must be addressed. A potential increase in adherence might have been achieved if health care professionals had provided personalized guidance on each patient’s physical activity level and had set specific goals. These strategies warrant further research to explore their impact on enhancing adherence to using activity trackers.

### Strengths and Limitations

The strengths of this study are the long measurement period of 1 year. This allowed for longitudinal analysis of how many patients recorded physical activity and how many discontinued their recordings. This insight further explains how activity trackers may function in clinical settings. Further strengths include the aspect of technical and operational feasibility, showing that the use of wearable activity trackers may involve an increased workload for the health care providers and require complex data management.

Limitations in the study include that we did not set cutoff values as to what constituted a valid week with physical activity measurement. This was decided because we wanted to explore the adherence to using the activity tracker. However, using >10 hours of valid day measurement has also been used by previous studies [22,40]. The hierarchical clustering analysis with the different wear-time patterns has a notable limitation, as it was conducted on a limited sample. This reduces the external validity of our study’s findings. Furthermore, the low number of patients per cluster group makes the comparison between these groups prone to biases and skewed data. Lastly, the definition of adherence used in this article may be misleading, as it uses the total amount of physical activity data that was returned. For example, technical issues related to synchronization may have led to missing physical activity data, thus not reflecting the actual use of the activity tracker.

### Implications

In order to increase the feasibility of wearable activity trackers, some optimizations are needed. First, data transfer should be automated, allowing for a “passive” collection of data. In the present study, patients had to use the MyDignio app and manually transfer data each week, which might have increased the burden on patients. Second, providing additional measurements beyond steps and heart rate would provide a more comprehensive overview of physical activity levels with time spent in different intensities and perhaps would increase adherence to using the activity tracker. Lastly, using wearable activity trackers, as demonstrated in this study, results in a vast amount of data. Considering the ethical perspectives of storing massive amounts of data, a structured and well-thought-out plan for how data are to be used should be implemented before physical activity data collection is initiated.

Future research should be based upon the recently published Wearable Activity Tracker Checklist for Healthcare (WATCH), which disposes a 12-point list of aspects to consider when implementing a wearable activity tracker in health care [43]. The checklist should be used in studies, preferably conducted in a real-world clinical setting. Additionally, “bring your own device” clinical studies may also hold promise as study design by representing a more accurate resemblance of real-world settings in which some patients already are owners of activity trackers [44]. Future research should incorporate qualitative methods to explore contributing factors behind the variations in wear-time patterns and adherence to using activity trackers.

### Conclusions

Despite the trendiness in both research on and commercial use of wearable activity trackers, long-term use of activity trackers had low to moderate feasibility over 1 year in patients with axSpA in low disease activity. The trial feasibility of using wearable activity trackers was low, while technical and operational feasibility were acceptable. Based on the wear-time patterns, we found that only 51% had consistently high activity tracker adherence. Future research should aim to ensure automated synchronization and investigate motivational factors influencing tracker adherence.

## Abbreviations

ASDAS-CRP: Axial Spondyloarthritis Disease Activity C-Reactive Protein Score
axSpA: axial spondyloarthritis
BASDAI: Bath ankylosing spondylitis disease activity index
BASFI: Bath ankylosing spondylitis functional index
TNFi: tumor necrosis factor inhibitor
